# Histomorphometric lung density evaluation of Immulina treatment using a murine influenza pneumonia model

**DOI:** 10.1111/iep.12493

**Published:** 2023-09-26

**Authors:** Floyd D. Wilson, Tahir M. Mir, Mohammad K. Ashfaq, Jin Zhang, Nirmal D. Pugh, Ikhlas A. Khan, Lanny W. Pace, Frederic J. Hoerr

**Affiliations:** ^1^ Mississippi Veterinary Research and Diagnostic Laboratory Mississippi State University Pearl Mississippi USA; ^2^ National Center for Natural Products Research, School of Pharmacy, Research Institute of Pharmaceutical Sciences University of Mississippi University Mississippi USA; ^3^ Department of BioMolecular Sciences, School of Pharmacy University of Mississippi University Mississippi USA; ^4^ Veterinary Diagnostic Pathology Fort Valley Virginia USA

**Keywords:** Immulina, lung density, mice, morphometrics, pneumonia

## Abstract

Histomorphometric lung density measurements were used to evaluate the effects of Immulina on mouse pneumonia. Mice were intra‐nasally exposed to H1N1 influenza virus at a dose of 5 × 10^4^ PFU/50 μL/mouse. Lung density was measured using the NIH ImageJ software program. Density values were compared to semiquantitative pneumonia severity scores. Lung photomicrographs were evaluated at 25‐×, 40‐× and 400‐× magnification. The study included viral inoculated controls (IC) and non‐inoculated controls (NC) and mice either treated or not treated with Immulina. Three doses of Immulina were included (25, 50 or 100 mg/kg) and administered using 3 protocols: prophylactic treatment (P), prodromal treatment (PD) and therapeutic treatment (TH) (note that in most of the evaluations of the data for the three treatment protocols were combined). Groups of mice were evaluated on days 3, 5, 7, 10 and 15 following exposure. The occurrence of “digital pneumonia” (DP) was defined as a density measurement above the 95% confidence limit of the corresponding NC values. A significant reduction in the occurrence of DP with Immulina treatment at the higher doses compared to IC was seen as early as day 3 and persisted up to day 15. There were also statistically significant dose‐variable reductions in lung density in response to Immulina. The study suggests early administration of Immulina (P or PD protocols) may enhance resistance against influenza‐induced viral pneumonia. A moderate correlation between pneumonia severity scores and lung density was observed for the 25‐× and 40‐× images (*R* = 0.56 and 0.53 respectively), and a strong correlation (*R* = 0.68) for 400‐× images.

## 1 INTRODUCTION

Microscopic morphometry is commonly used for the quantification of pathological processes.^1^, ^2^, ^3^ A histomorphometric method using density measurements to quantify bone marrow cellularity was described in our previous research.^2^ In this method, the term density specifically refers to the mean grey value for a selected image. We subsequently adapted this density approach for the histologic measurements of severity and occurrence in experimental murine viral pneumonia.^3^ The pulmonary density analysis method uses the free downloadable NIH ImageJ software program in conjunction with a standard microscope with an attached camera.

Immulina is a commercially available botanical dietary supplement in the United States and Europe that is a standardized extract of *Limnospira*. Previous research indicates that Immulina exhibits immune‐enhancing effects and could be useful for altering the host antiviral immune response. For example, oral administration of Immulina to mice (30 days pre‐ and 21 days post‐infection) has been shown to decrease the severity of H1N1 influenza viral pneumonia as reflected by significant reductions in weight loss, clinical signs of disease and histopathology severity scores.^4^ The protective effect of Immulina in mice against influenza infection could be mediated by activation of the host immune system since both animal studies^5^ and human trials^6^, ^7^ provide evidence that this product enhances various immune defence mechanisms.

In the current communication we report the application of the pulmonary density method to quantify the effects of oral administration of Immulina on mouse pneumonia. The lung histology sections analysed were archived samples from in vivo mouse efficacy studies evaluating the potential utility of Immulina to enhance antiviral immune resilience against influenza infection.

## 2 METHODS

### 2.1 Materials

The Immulina™ test material used is a crude extract of *Arthrospira/Limnospira* that was provided by ChromaDex (lot 2290020). Taxonomic identification of the raw material was previously performed using morphological examination and sequence analysis and determined to be *Limnospira* fusiformis.^8^ Immulina is standardized using a bioassay employing THP‐1 human monocytes that quantitates potency to ensure batch to batch consistency of activity.

For the mouse studies, the desired dose of Immulina was administered in a volume of 200 μL of nanopure sterilized water by oral gavage.

### 2.2 Mouse studies

The study used C57BL/6 male and female mice (Envigo). An approximately equal number of male and female mice (20–22 gram) were included in the study. However, to get the greatest number of mice for statistical evaluation of density effects, the data for sexes were combined. Preliminary examinations did not disclose major differences between males and females for Immulina density effects.

Immulina was evaluated in three mouse protocols of resilience against influenza A virus (H1N1): (1) prophylactic (P)—mice were fed once daily (by oral gavage) with Immulina for only during the 2 weeks prior to viral infection, (2) prodromal (PD)—administration of Immulina or vehicle was initiated on the day of viral infection (2 h post‐infection) and continued once daily through day 15, and 3) therapeutic (TH)—Immulina or vehicle was administered starting on day 3 post‐viral infection (at the first appearance of signs of infection) and continued once daily through day 15.

For each mouse protocol, five groups were evaluated. Groups 1–3 consisted of administering three different doses of Immulina (100, 50 and 25 mg/kg) with *n* = 38–50 mice per group. Group 4 were infected controls (IC, *n* = 39–45) and given vehicle only. Mice in groups 1–4 were intranasally infected with mouse‐adapted influenza A virus (H1N1) strain A/PR/8/34 (ATCC VR‐1469) in EMEM media. The viral dose was 5 × 10^4^ PFU/50 μL/mouse and represented the optimal amount for a non‐lethal infection model. The fifth group of mice (*n* = 20–40) served as noninfected controls (NC) ‐ they were administered water and were not infected with virus.

Mice were evaluated at 3, 5, 7, 10 and 15 days following virus exposure. At each time point 20% of the mice were sacrificed and a portion of lung tissue was collected aseptically and placed in neural buffered formalin for histology slide processing. Standard 5‐μm‐thick H&E‐stained sections were used for both histology and morphometric studies. The influence of Immulina treatment on the establishment and progression of viral pneumonia in mice was evaluated histologically in a total of 617 lung samples. Microscopic evaluations were performed using an Olympus CX31 microscope (Olympus Corporation, Breinigsville, PA). Photomicrographs were taken with an AmScope MU1403 camera (AmScope, Irvine, CA) using the associated software‐based program. A detailed description of the mouse pneumonia model was published previously by other investigators.^8^

### 2.3 Morphometrics

The presence of pneumonia was first diagnosed using routine histopathological criteria.

The morphometric approach used for quantifying lung cellularity using density measurements was reported previously.^3^ Briefly, the lung sections were photographed at either 25‐× (1 image), 40‐× (1 image) and 400‐× (3 images) magnifications. For each histology section the entire lung area present at 25‐× and 40‐× were outlined and measured. Three microscopic fields from the most affected regions were used for the 400‐× evaluations, each field measuring 15,510 μm^2^ with a total area of 46,530 μm^2^.

The coloured photographic images were converted to a binary format using the downloadable National Institutes of Health (NIH) ImageJ software program (https://imagej.nih.gov/ij/download.html). Digital features of the binary images were then calculated using the image analysis software. The mean grey values and integrated densities were determined using the “Analyse‐Measure” command of the menu. Integrated density represents the product of the area and mean grey value. Unless otherwise stated the term mean density as used in this report refers to the mean grey value of the image. The occurrence of “morphometric pneumonia” (MP) was arbitrarily defined as a density value that was above the upper 95% confidence interval for the corresponding NC control value and at a particular magnification.

Semiquantitative pneumonia severity scoring of the images was also performed. A six‐level scoring system for grading pneumonia was used in which: 0 = absent, pneumonia not observed; 1 = minimal severity (pneumonia barely detectable); 2 = mild; (a small area and mild inflammatory cell infiltration) 3 = moderate (an extensive area of typically multifocal involvement with numerous inflammatory cells and exudation); 4 = marked (a more or less diffuse area of involvement with extensive inflammatory exudate); and 5 = severe (diffuse lung involvement with massive inflammatory exudate sometimes including adenomatosis). When uncertainty existed between scoring groups in assigning a value, half values were given.

The occurrence of pneumonia when using density measurements was arbitrary defined as “digital pneumonia” (DP). This represents a density measurement above the upper 95% confidence limit of the corresponding NC group.

### 2.4 Statistical evaluations

Statistical analysis utilized the GraphPad Prism version 9 software program (GraphPad Software, Inc. San Diego, CA). Statistical differences between the groups were determined using the Kruskal‐Wallis ANOVA test and the Dunn's test for multiple comparisons. Statistical differences in pneumonia occurrence utilized the Fisher's exact test. Correlation coefficients were calculated using the Pearson test. The Pearson test was used for calculation of correlations. The data presented in the graphs depict the mean ± one standard deviation.

## 3 RESULTS

**FIGURE 1:**
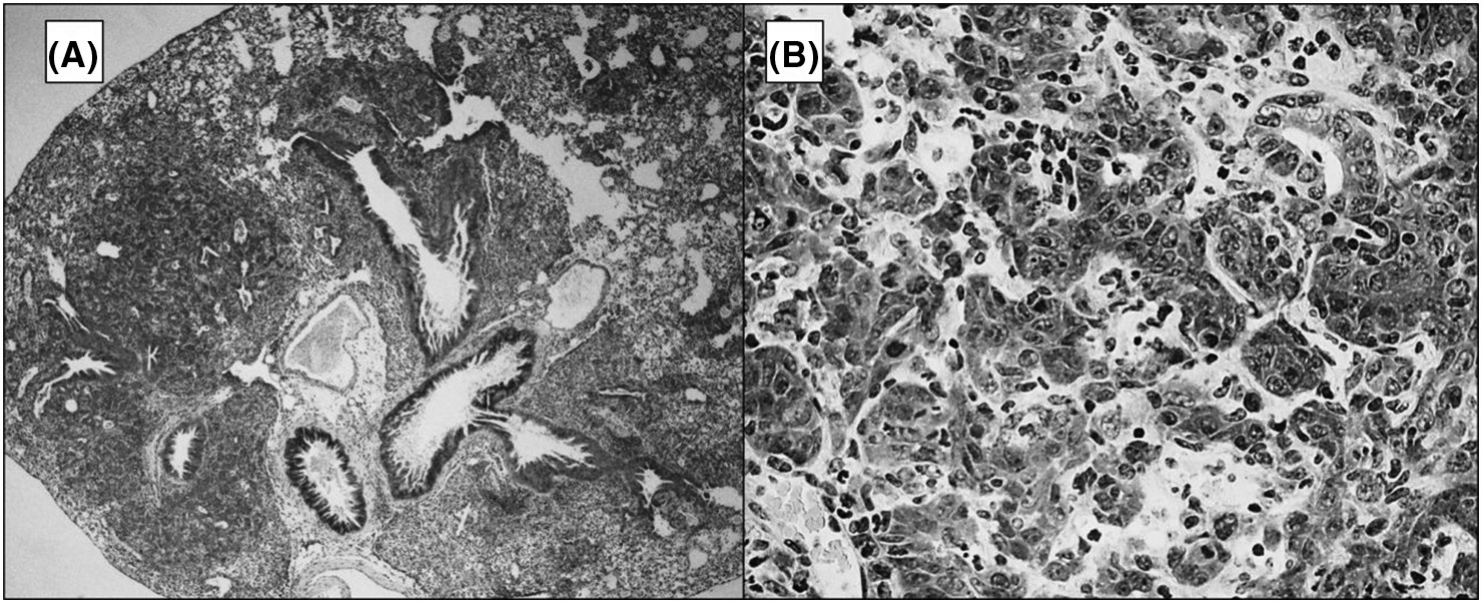
Histological examples of pneumonia from a mouse from the IC group. 1A Lung section shown at 40‐×. 1B same IC lung shown at 400‐×.

The pneumonia produced was similar to that previously described for this virus.^9^ Histological examples of pneumonia from an IC group mouse are shown in Figure 1. The pneumonia was characterized microscopically by thickening and hypercellularity of the alveolar walls and flooding of the alveolar lumina with mainly mononuclear inflammatory cells (Figure 1A). Cuboidal metaplasia of the alveolar pneumocytes was sometimes present resulting in an adenomatous appearance (Figure 1B). The consolidation collectively resulted in an increased lung density. The severity score for the pneumonia example was 3.5, while the morphometric mean densities were 169 and 121, respectively, for the two magnifications. Detailed results for routine histopathology evaluations are the subject of a separate report. However, the mean severity scores for pneumonia are given in Table 1.

**TABLE 1:** Average pneumonia severity scores.

PF group																									
Day	Day 3					Day 5			Day 7					Day 10					Day 15						
Treatment	NC	IC	100	50	25	100	50	25	NC	IC	100	50	25	NC	IC	100	50	25	NC	IC	100	50	25		
Pneumonia score	1.0	1.4	1.4	1.4	2.3	1.8	2.5	2.4	0.0	3.8	2.9	2.8	2.8	2.0	3.0	0.8	0.0	2.3	1.0	3.3	2.0	1.3	0.9		
PM group																									
Day	Day 3					Day 5					Day 7					Day 10					Day 15				
Treatment	NC	IC	100	50	25	NC	IC	100	50	25	NC	IC	100	50	25	NC	IC	100	50	25	NC	IC	100	50	25
Pneumonia score	0.5	3.0	0.4	1.6	1.1			1.1	1.9	2.5	0.0	3.4	0.0	1.9	2.6	0.3	2.2	2.1	2.9	0.6	0.8	2.0	1.5	1.8	0.2
PDM group																									
Day	Day 3					Day 5					Day 7					Day 10					Day 15				
Treatment	NC	IC	100	50	25	NC	IC	100	50	25	NC	IC	100	50	25	NC	IC	100	50	25	NC	IC	100	50	25
Pneumonia score	0.3	1.5	1.8	2.4	2.1	0.5	2.8	2.5	2.5	2.1	0.3	3.6	2.4	2.5	2.6	1.5	3.4	0.6	1.6	2.8	1.3	3.8	1.1	1.0	1.6
PDF group																									
Day	Day 3					Day 5					Day 7					Day 10					Day 15				
Treatment	NC	IC	100	50	25	NC	IC	100	50	25	NC	IC	100	50	25	NC	IC	100	50	25	NC	IC	100	50	25
Pneumonia score	0.9	1.0	0.4	1.2	1.5	0.0	2.0	1.1	1.7	1.7	M	2.7	2.0	1.7	3.0	0.1	3.7	1.0	1.8	2.1	0.3	3.2	1.2	0.6	2.0
THF group																									
Day	Day 3					Day 5			Day 7					Day 10					Day 15						
Treatment	NC	IC	100	50	25	100	50	25	NC	IC	100	50	25	NC	IC	100	50	25	NC	100	50	25			
Pneumonia score	0.0	3.4	2.7	2.6	2.8	2.8	1.6	2.7	0.5	3.3	3.1	1.9	2.8	0.0	2.6	2.9	2.8	2.2	0.3	1.1	1.8	1.9			
THM group																									
Day	Day 3					Day 5			Day 7					Day 10					Day 15						
Treatment	NC	IC	100	50	25	100	50	25	NC	IC	100	50	25	NC	IC	100	50	25	NC	IC	100	50	25		
Pneumonia score	0.6	2.9	2.0	1.8	1.8	1.2	2.1	1.2	0.0	2.4	2.0	2.1	2.2	0.3	3.4	2.9	3.3	2.5	0.0	2.1	1.5	1.1	2.7		

**FIGURE 2:**
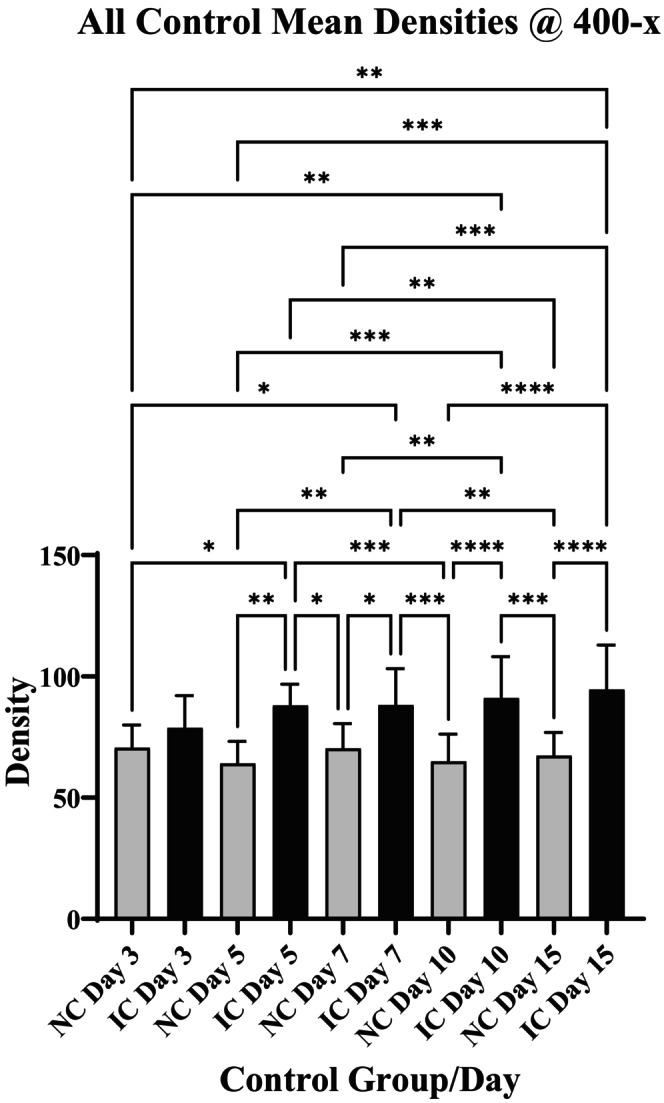
Comparison of density measurements on various days for NC and IC controls using 400‐× Images. There is a significant elevation in the IC values relative to NC on all but day 3. The data presented in the graphs depict the mean ± one standard deviation. Asterisk incidates strength of significance.

The results comparing density measurements on various days for NC and IC controls using the 400‐× images are depicted graphically in Figure 2. There is a significant elevation in the IC values relative to NC on all but day 3. Although there were significant differences between the NC and IC groups at all 3 magnifications, they were most pronounced using the 400‐× measurements. The number of asterisks indicates the strength of significance.

**FIGURE 3:**
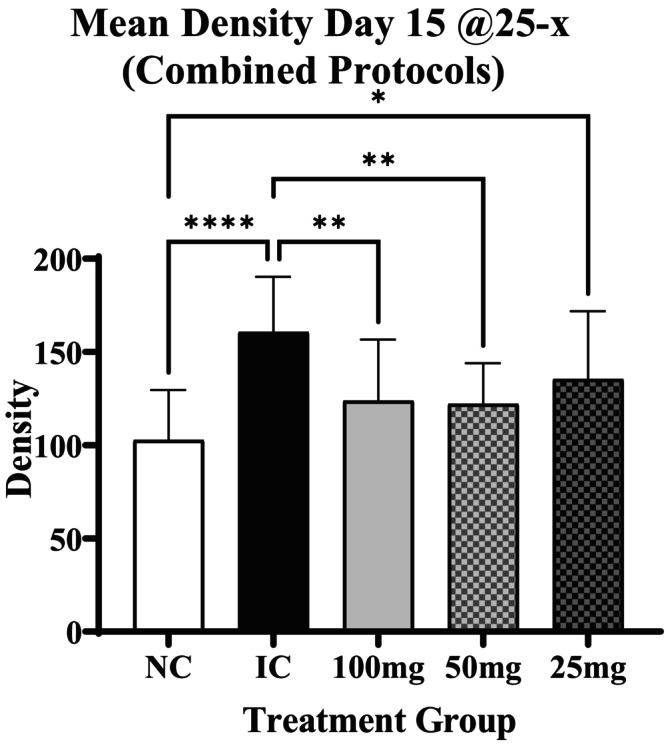
Therapeutic density effects of Immulina for 25‐× measurements on day 15. Significant Immulina therapeutic effects were seen at the 100 mg and 50 mg doses on day 15 at 25‐× and after combining the protocol data. The data presented in the graphs depict the mean ± one standard deviation. Asterisk indicates strength of significance.

The most numerous significant Immulina therapeutic effects were seen for the 25‐× measurements on day 15 after combining the data from all three protocols (Figure 3). In this example, a significant reduction in lung density relative to IC is evident for the 100 and 50 mg/kg doses, but not for 25 mg/kg treatment (Figure 3).

**FIGURE 4:**
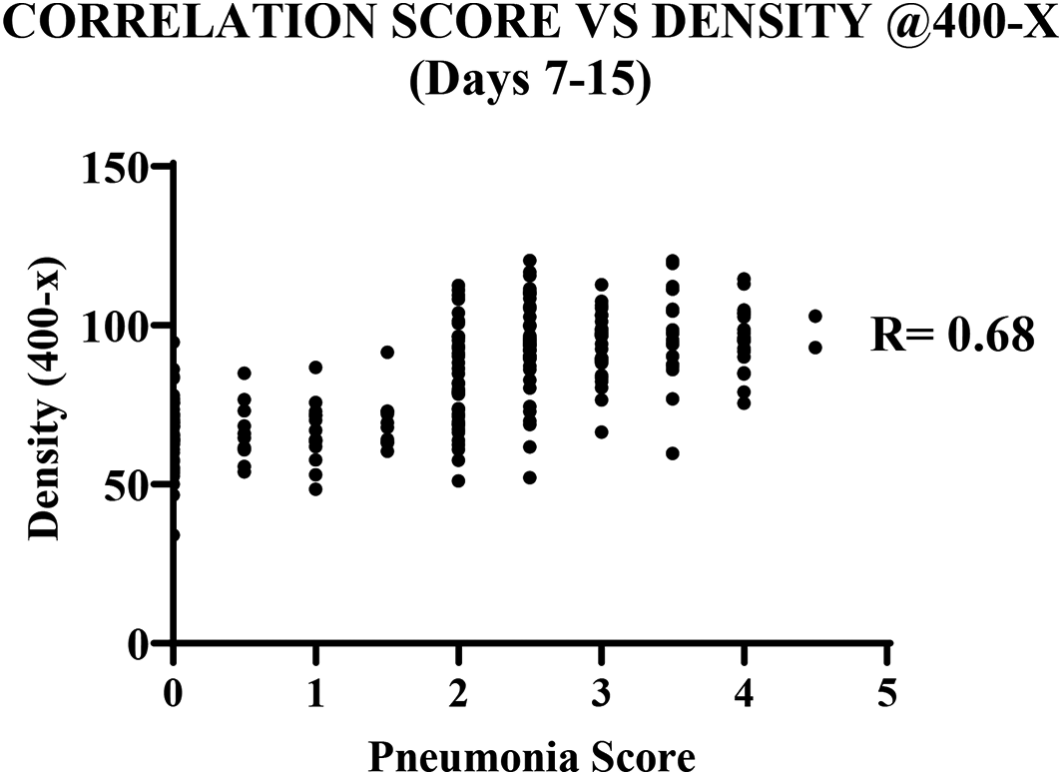
Correlation between pneumonia severity score and density (400‐×, days 7–15).

The strength of correlation between the results for pneumonia severity scoring and mean density measurements were compared for the various magnifications evaluated. The correlations were calculated using the treatment data from days 7, 10 and 15; representing times when pneumonia was most pronounced. While the strength of the correlation coefficient when using the 25‐× and 40‐× density data were moderate (*r* = 0.56 and *r* = 0.53 respectively), there was a strong correlation when using the 400‐× evaluation data (*r* = 0.68). The correlation data for the 400‐× evaluation is presented in Figure 4.

**FIGURE 5:**
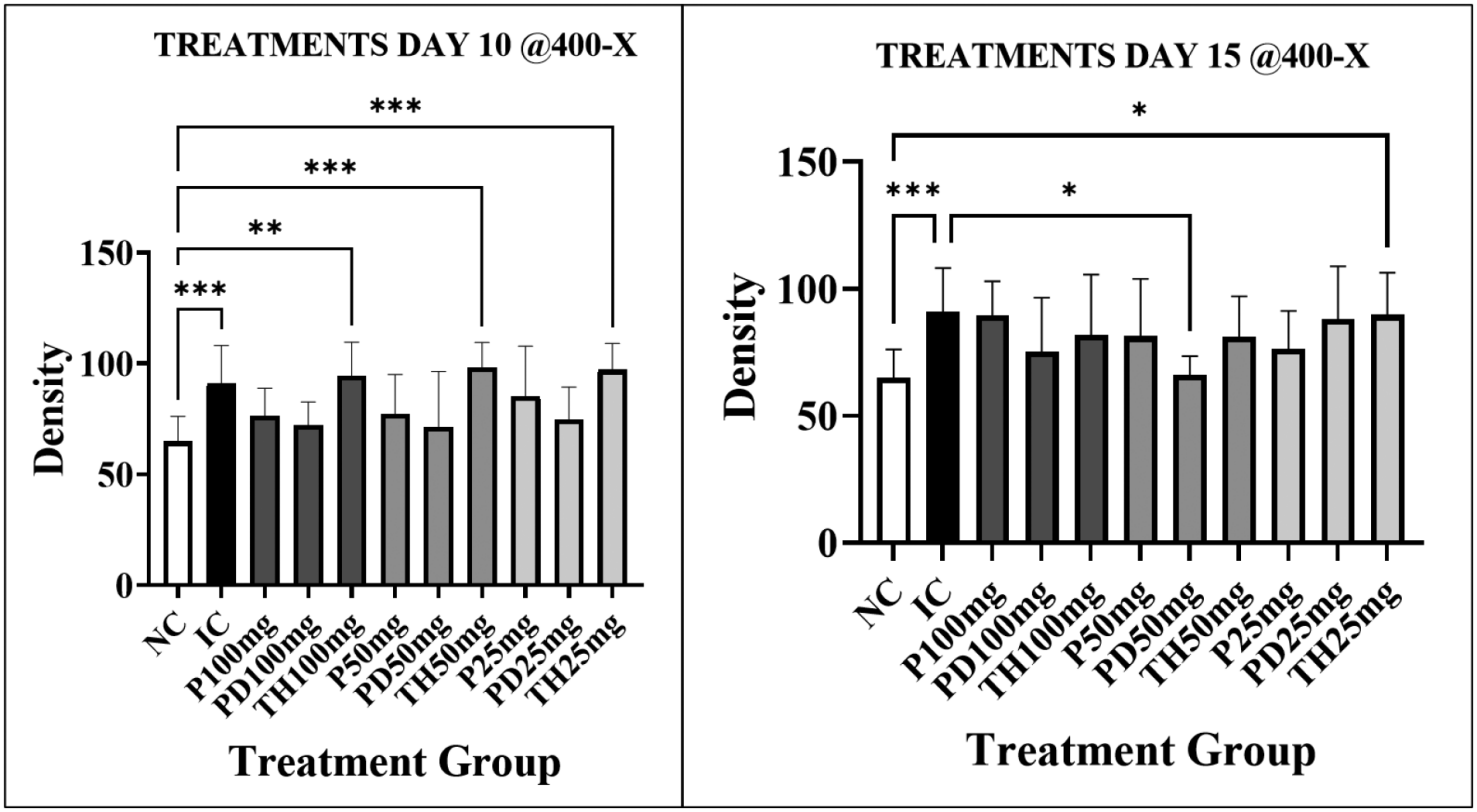
Effects of Immulina therapy and delivery protocol on lung density on days 10 and 15. The data presented in the graphs depict the mean ± one standard deviation. Asterisk indicates strength of significance.

There were other density findings that, although not significant, were suggestive of a reduction in density occurring in response to Immulina administration. For example, the effects of Immulina treatment protocols on density using the 400‐× measurements are depicted graphically for days 10 and 15 in Figure 5. Although mostly not significant, there is an overall suggestion of therapeutic effects on density when using the P and PD protocols, but not for the TH group. The apparent reduction of TH Immulina therapeutic effects was most pronounced on day 10 (Figure 5) where all doses were significantly higher as compared to NC, but very similar to IC values. However, these 400‐× density differences were less apparent on day 15 (Figure 5). Also, the density effects of dosing schedule for treatment for the 3 protocols were less evident for the 25‐× and 40‐× measurements (data not shown).

**FIGURE 6:**
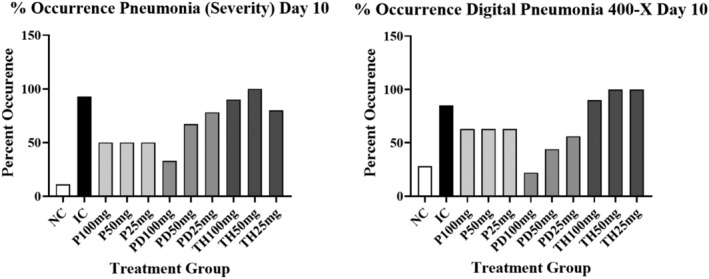
The effects of Immulina dose and treatment protocol on pneumonia occurrence using severity scoring shown on the left or density measurements shown on the right (Day10). The occurrence of pneumonia for the density measurements was arbitrary defined as “digital pneumonia” representing a density measurement above the upper 95% confidence limit of the corresponding NC group. The data presented in the graphs depict the mean ± one standard deviation.

**FIGURE 7:**
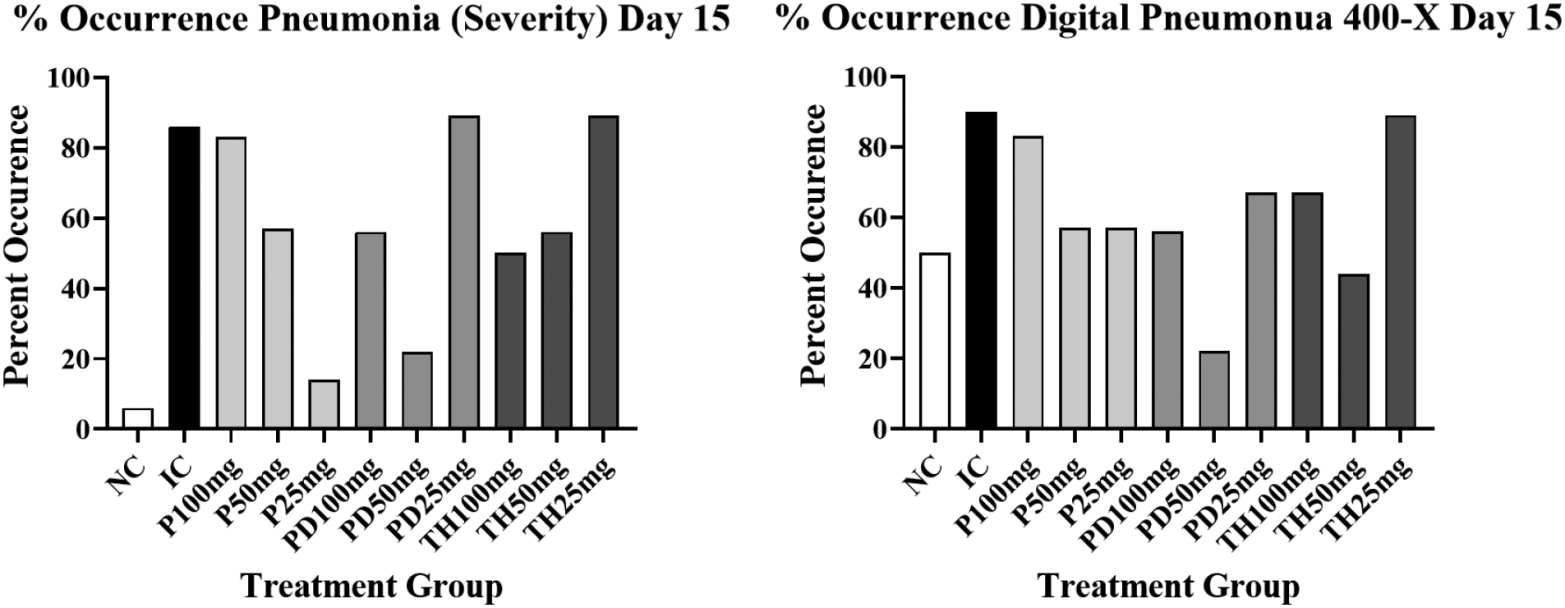
The effects of Immulina dose and treatment protocol on pneumonia occurrence using severity scoring shown on the left or density measurements shown on the right (Day15). The occurrence of pneumonia using density measurements was arbitrary defined as “digital pneumonia” which represents a density measurement above the upper 95% confidence limit of the corresponding NC group. The data presented in the graphs depict the mean ± one standard deviation.

The results comparing the % pneumonia occurrence using either severity scoring or “digital pneumonia” measurements (morphometry @400‐×) are depicted graphically in Figures 6 and 7. Statistical comparison for pneumonia occurrence results are given in Tables 2 and 3.

**TABLE 2:** Statistical evaluation of Immulina dose on pneumonia occurrence using scoring or morphometrics.

Analysis of occurrence (Fisher's exact test)							
Method		Severity score			Morphometrics 400‐×		
	Dose	100 mg	50 mg	25 mg	100 mg	50 mg	25 mg
Control	Day						
NC	3	0.0055	0.0017	0.010	>0.9999	>0.9999	0.0305
IC		0.1028	0.1028	0.2581	0.0495	0.0268	0.7761
NC	5	0.0006	0.1551	<0.0001	0.0113	0.0095	0.0011
IC		0.0309	<0.0001	0.2204	0.0159	0.0175	0.1395
NC	7	<0.0026	<0.0001	<0.0001	0.2133	0.0305	0.0053
IC		0.0113	0.0243	0.0504	0.0473	0.3718	>0.9999
NC	10	0.0118	0.0002	0.0002	0.0664	0.0069	0.0051
IC		0.6337	0.0764	0.0764	0.0047	0.3265	0.5007
NC	15	0.0065	0.1093	<0.0001	0.3483	0.7555	0.1247
IC		0.0054	0.0002	0.1878	0.0776	0.0007	0.1506

*Note*: Shade indicates significance.

**TABLE 3:** Statistical evaluation of treatment protocol effects on pneumonia.

Analysis of occurrence (Fisher's exact test)							
Method		Severity score			Morphometrics 400‐×		
Protocol		P	PD	TH	P	PD	TH
Control	Day						
NC	3	0.0032	0.0118	0.0114	0.7417	>0.9999	0.2261
IC		0.3772	>0.9999	0.1664	0.1564	0.0505	0.6232
NC	5	<0.0001	0.0002	0.0001	0.0061	0.0006	0.0337
IC		0.2072	0.1215	0.0702	0.3723	0.1164	0.3992
NC	7	<0.0001	0.0005	<0.0001	0.0578	0.0122	0.0004
IC		0.0085	0.0117	0.1946	0.3564	0.7585	0.4395
NC	10	0.0100	<0.0001	<0.0001	0.0278	0.5271	<0.0001
IC		0.0012	>0.9999	>0.9999	0.1867	0.0016	0.4077
NC	15	0.0043	<0.0001	0.0001	0.5118	>0.9999	0.355
IC		0.0203	>0.9999	0.1141	0.0670	0.0023	0.0833

*Note*: Shade indicates significance.

The effects of treatment on pneumonia occurrence are presented in Figures 6 and 7 for days 10 and 15 respectively. On day 10 there is a strong and similar suggestion of a reduction in pneumonia occurrence in response to Immulina therapy using either severity scoring or density measurements @ 400‐× (Figure 6). This is apparent for all doses using the P protocol, but mostly at the highest dose with the PD protocol. However, no evidence of an occurrence treatment response is seen using the TH protocol. Generally similar results were seen on day 15 (Figure 7). However, some indication of Immulina effects is present for TH protocol on day 15 for the severity data but less so for the density measurements.

Statistical comparisons for the effect of Immulina or treatment protocol on pneumonia occurrence are shown in Tables 2 and 3. The density measurements made @400‐× were used as this magnification revealed the greatest overall differences. Note that the possible influence of when treatment was initiated was ignored in the dose evaluations (ie P, PD and TH subgroup data was combined). Statistical results for pneumonia occurrence comparing therapy protocols effects are given in Table 3.

Numerous Immulina treatment‐associated statistical differences from the corresponding NC or IC are apparent. A significant reduction in “digital pneumonia” occurrence was observed for the 100 and 50 mg/kg treatments as early as day 3 (Table 2). This was reflected by a reduction in density relative to the IC group. However, this difference was not apparent using histologic severity scoring (Table 2).

In most cases using scoring, pneumonia occurrence in Immulina groups was significantly higher than the NC group on all days and doses (Table 2). However, fewer significant differences from the IC group which indicates an Immulina therapeutic effect are seen with scoring. On all days except day 15, the digital pneumonia occurrence of the high 100 mg/kg dose was significantly lower than the IC group.

Statistical evaluations of treatment protocols are shown in Table 3. A significant reduction in pneumonia occurrence using scoring was evidenced on days 7 through 15 for the P protocol. A significant pneumonia occurrence reduction was only present on day 7 with the PD protocol. However, no significant reduction in pneumonia occurrence using scoring was produced using the TH protocol (Table 3). The differences in treatment initiation protocols were less apparent using the density method as significant therapeutic effects were only observed for the PD protocol on days 10 and 15. It should be noted that for protocol evaluations the data for different doses were combined.

## 4 DISCUSSION

Pulmonary histomorphometrics was used to evaluate the influence of Immulina oral administration in mice on both establishment and progression of viral pneumonia. Immulina is a dietary supplement extract from *Arthrospira/Limnospira* and research indicates that this botanical product has immune modulatory properties which may provide host resilience against respiratory viral infection.^4^, ^10^, ^11^

A significant reduction by Immulina treatment was shown in both occurrence of DP and mean density of infected lungs. In addition, a strong correlation was observed between the results for semiquantitative pneumonia severity scoring and mean lung density measurements (*R* = 0.73 at the 400‐× magnification).

Our previous research demonstrated that oral administration of Immulina in mice (30 days pre‐ and 21 days post‐infection) substantially reduced various pathological parameters due to influenza A virus (H1N1) infection.^4^ However, since Immulina was administered throughout the study (pre‐ and post‐infection), the question remained as to whether the observed antiviral resilience was a prophylactic, prodromal and/or therapeutic effect. To address this question, the current study evaluated the effectiveness of Immulina in three separate protocols of resilience against influenza virus infection (P, PD and TH). The study indicated that the timing for initiation of Immulina treatment may be important for successful therapy, regardless of dose. Using the 400‐× data on day 10, pneumonia severity appeared somewhat reduced by the P and to a lesser extent the PD protocols, but not after symptoms were well established (TH protocol). However, this therapy timing effect was less apparent on day 15 and not detectable when using the 25‐× or 40‐× data. Thus, while Immulina effects appear most significant when given early, some suggestion of therapeutic effects of the TH protocol was apparent on day 15. This suggests that the effects of Immulina evaluated in this protocol may be delayed.

The density results using 25‐× and 40‐× images were less strongly correlated with histologic severity scoring compared to the 400‐× results. This likely reflects a “dilution effect” owing to the incorporation of larger normal lung regions in photomicrographs taken at lower magnifications. For example, the entire lung section area was present for density measurements at 25‐× which typically includes extensive normal lung regions. This allows for estimation of overall pneumonia involvement reflecting both intensity and lung area involvement. In contrast, the 400‐× density measurements almost exclusively reflect the intensity of pneumonia in the affected lung regions. Thus, measurements at two or more magnifications can potentially provide different types of useful quantitative information on pneumonia involvement.

Digital density measurement also provides a quantitative method for validation of pneumonia severity scoring. The correlation of lesion scoring results with lung density measurements facilitates the establishment of normal ranges and the standardization of severity scoring between different observers. The image density method provides a simple, rapid and inexpensive method for quantification of pneumonia parameters using routine histology sections, standard microscopy and a free downloadable software program.

## FUNDING INFORMATION

This research was partly funded by the Office of Dietary Supplements and National Center for Complementary and Integrative Health of the National Institutes of Health under Award Number U19AT010838. The content is solely the authors' responsibility and does not necessarily represent the official views of the National Institutes of Health. Additional funding was also provided by a grant from the USDA, Agricultural Research Service Specific Cooperative Agreement No. 58–6060‐6‐015.

## CONFLICT OF INTEREST STATEMENT

NDP and IAK acknowledge financial interest in Immulina. All other authors declare that they have no known competing financial interests or personal relationships that could have appeared to influence the work reported in this paper.

## ANIMAL STUDIES (HUMANE CARE AND USE OF ANIMALS)

The study followed protocols approved by the Institutional Animal Care and Use Committee at the University of Mississippi.
